# The Impact of Proactive Personality on Career Decision-Making Self-Efficacy: The Role of AI Acceptance and Innovation Skills

**DOI:** 10.3390/bs15040538

**Published:** 2025-04-16

**Authors:** Kunji Li, Jeffery D. Houghton, Siyu Chen, Xuan Li, Danyang Li, Wenchi Zou

**Affiliations:** 1 College of Management, Guangdong University of Science and Technology, Dongguan 523083, China; 2 John Chambers College of Business and Economics, West Virginia University, Morgantown, WV 26506, USA; 3 School of Business, Macau University of Science and Technology, Macau

**Keywords:** proactive personality, generative artificial intelligence acceptance, career-related decision-making self-efficacy, innovation competencies

## Abstract

This study aimed to investigate the relationship between proactive personality and career-related decision-making self-efficacy, with generative artificial intelligence acceptance serving as a mediating factor. Additionally, the study examined the moderating effect of innovation competencies on this mediation pathway, utilizing a moderated mediation framework. The study included 501 university students from Guangdong Province, China, who completed validated measures of proactive personality, career-related decision-making self-efficacy, generative artificial intelligence acceptance, and innovation competencies. Data analysis was conducted using SPSS (Version 26) and the PROCESS macro to evaluate mediation, moderation, and moderated mediation effects. Moreover, confirmatory factor analysis was performed using Amos (Version 28.0) to assess the validity of the measurement models. The results indicate that proactive personality exerts an indirect effect on career-related decision-making self-efficacy via generative artificial intelligence acceptance. The strength of this indirect effect was moderated by innovation competencies, indicating that the relationship is stronger at higher levels of innovation competencies. These findings contribute to a more nuanced understanding of the relationship between proactive personality and career-related decision-making self-efficacy, particularly within the context of technological adaptability.

## 1. Introduction

Currently, the employment environment for college students is undergoing rapid and significant changes. An important factor affecting these changes is the rapid development of artificial intelligence technology, such as tools like ChatGPT. The development of this technology has resulted in a degree of uncertainty in the job market, as various professions are being reshaped or disrupted by these innovative technologies. In order to succeed in today’s demanding professional landscape, young individuals need to make informed career choices and engage in appropriate behaviors that guide them in selecting the right career path. Career-related decision-making, an important turning point for individuals transitioning from school to the workforce, not only influences the success of individual careers, but also profoundly affects the overall dynamics and structural layout of society (Arbona et al., 2024). However, in this complex and changing context, college students often encounter numerous difficulties in the career-related decision-making process, including but not limited to asymmetric information, ambiguous career perceptions, a lack of self-understanding, and uncertainty about the external environment (Kulcsár et al., 2020), which collectively render employment-related decision-making a challenging and stressful endeavor (Lee et al., 2022).

Research on occupational psychology and organizational behavior has shown that a proactive personality can increase self-efficacy in employment-related decision-making (Kim & Park, 2017; Tanau & Salim, 2020). Proactive individuals are often viewed as more confident and motivated in exploring, choosing, and achieving their professional objectives, thereby enhancing their ability to arrive at well-considered career choices (He et al., 2021; N. Wang et al., 2023). Despite its importance, the significance of generative artificial intelligence acceptance—encompassing individuals’ attitudes, intentions, and perspectives toward new technologies—has not been thoroughly examined in relation to career-related decision-making. This is because most of the existing literature focuses on traditional career environments, paying scant attention to how technological advancements—especially as part of the developing landscape of generative artificial intelligence—impact the vocation selection process.

Given the rapid technological advances and widespread adoption of generative artificial intelligence, coupled with the changes occurring in the job market (Frank et al., 2019; Zarifhonarvar, 2024), college students are now required to show greater flexibility, a commitment to continuous learning, and innovation competency, in addition to possessing strong professional knowledge and skills (Shen & Zhang, 2024). However, there is little existing research on the role that innovation competencies play in the link between generative artificial intelligence acceptance and beliefs regarding personal competencies related to vocational determination. Therefore, it is of great theoretical and practical significance to incorporate generative artificial intelligence acceptance and innovation competencies into the research framework examining the impact of a proactive personality on one’s sense of self-efficacy in job choice capabilities, and to explore the role played by generative artificial intelligence acceptance and innovation competencies in this context.

## 2. Literature and Hypothesis

### 2.1. Proactive Personality and Career-Related Decision-Making Self-Efficacy

According to Bateman and Crant (1993), proactive personality is a dispositional trait defined by the propensity to take the initiative and bring about change in the environment. According to the proactive personality hypothesis, individuals who exhibit high levels of proactivity are more inclined to pursue their goals and persevere in the face of difficulties. These individuals are resilient and action-oriented, thereby equipping them to handle the challenges of making difficult professional decisions. Bandura (1977) introduced the concept of “self-efficacy”, referring to an individual’s belief in their ability to implement the necessary measures to accomplish specific performance goals. The significance of self-efficacy in achieving success in various life domains, including career-related decision-making, is highlighted by Bandura’s social cognitive theory (Wood & Bandura, 1989). Career-related decision-making self-efficacy is a specific type of self-efficacy that involves the dimensions of gathering career-related information, setting career-focused goals, and engaging in career-oriented problem-solving (Betz et al., 1996).

Social cognitive theory provides theoretical support for a possible relationship between proactive personality and career-related decision-making self-efficacy. Specifically, social cognitive theory suggests that people are subject to both proactive and reactive control mechanisms (Bandura, 1991). People can motivate themselves through proactive or anticipatory control processes by setting standards for their performance that create a disequilibrium that mobilizes effort to reach the goal (Bandura, 1991). In contrast, reactive control processes act as a more passive response to negative feedback (Bandura, 1991). In the current context, students with a proactive personality are more likely to be subject to proactive control processes resulting in career-related information gathering, goal-setting, and problem-solving, thus enhancing career-related decision-making self-efficacy. Less proactive students will be more subject to reactive control processes, and will likely experience lower levels of career-related decision-making self-efficacy.

The dual-process theory of cognition (Evans & Stanovich, 2013; Kahneman, 2011) provides additional complimentary support to this theoretical assertion. Dual process theory suggests that people engage in two types of thinking: rapid, autonomous, and intuitive (type 1) vs. slow, deliberate, and reflective (type 2) (Evans & Stanovich, 2013). Oftentimes, individuals are cognitive misers, defaulting to intuitive thinking (type 1) rather than engaging in reflective reasoning (type 2) involving information-gathering and problem-solving (Kahneman & Frederick, 2002). In the current study, students with a proactive personality were found to be more likely to engage in more deliberate reflective and rational processes (type 2) in approaching career-related decision-making, rather than defaulting to autonomous and intuitive processes (type 1), thereby enhancing career-related decision-making self-efficacy.

In addition to these theoretical rationales, there has been considerable empirical research interest in the connection between proactive personality traits and perceived self-efficacy regarding vocational determination. A proactive personality is characterized by an inclination to take the initiative, perseverance in effecting significant changes, and a strong work ethic to achieve success in various life domains, including career choices (Brown et al., 2006). Research consistently suggests that proactive personalities are associated with increased self-efficacy in the career-related decision-making process (Zhou et al., 2018; N. Wang et al., 2023; Duru & Söner, 2024). Preston and Salim (2019) further explained that a proactive personality reinforces self-efficacy beliefs by fostering a sense of control and a readiness to tackle work difficulties. Zhou et al. (2018) described perceived self-competence as a reflection of an individual’s belief in their proficiency to effectively execute the steps involved in exploring employment options and making job/role choices, which is essential when transitioning from school to the workforce. Additionally, Kim and Park (2017) stressed that by encouraging a proactive attitude toward career planning, a proactive personality directly influences career-related decision-making processes. This approach entails information-gathering, networking, and remaining up-to-date on career options to build strong self-efficacy beliefs regarding professional trajectory determination. According to a wealth of research, a proactive personality and self-efficacy are crucial factors in making well-informed career path decisions. Based on interactionist and social cognitive theories (Bandura, 1991), individuals possessing a proactive personality utilize their initiative and resilience to strengthen their self-efficacy beliefs in the context of career-related decision-making. Building on these theoretical foundations and supported by empirical evidence, we posit the following hypothesis:

**Hypothesis** **1** **(H1).** *Proactive personality is positively related to career-related decision-making self-efficacy.*

### 2.2. Proactive Personality and Generative Artificial Intelligence Acceptance

Previous empirical research results reveal that people often hold a traditional view that AI-assisted decision systems cannot be trusted due to reduced human-to-human interaction, which makes people perceive a lack of transparency, along with unfamiliarity with such tools, and concerns about low reliability and accuracy (Calluso & Devetag, 2024; Glikson & Woolley, 2020; Horodyski, 2023). However, as AI technologies have developed, they have been extensively used in college students’ academic and career navigation (Page & Gehlbach, 2017; Zhai et al., 2021; Ng et al., 2024; Yu et al., 2023), in mentorship programs of medical students (Burke & Gwillim, 2024), and in self-regulated learning and science education (Ng et al., 2024). Furthermore, scholars have found that the ethical use of AI can help college students enhance their levels of self-esteem, self-efficacy, and self-control (Rodríguez-Ruiz et al., 2025), indicating that AI is becoming increasingly critical and reliable in assisting college students’ growth, whether this be in terms of their career development or their psychological maturity. Therefore, it is reasonable to argue that AI is one of the most critical and valuable information resources for college students. Previous research suggests that individual personality traits can influence technology adoption. For example, individuals with distinct personality traits (e.g., the Big Five personality traits) exhibit varying preferences regarding instant messaging and metaverse technologies (Barnett et al., 2015; Ehrenberg et al., 2008; Sowmya et al., 2023). Building on this foundation, the present study aims to extend this line of inquiry by exploring the relationship between proactive personality and generative artificial intelligence acceptance through the theoretical lens of the Technology Acceptance Model (TAM) and the Unified Theory of Acceptance and Use of Technology (UTAUT).

The Technology Acceptance Model (TAM), a well-established theoretical framework in the study of information technology adoption, posits that users’ willingness to adopt new technologies is primarily determined by two critical factors, namely, perceived usefulness and perceived ease of use (Davis, 1989). This model serves as a robust foundation for investigating the mechanisms driving individuals’ acceptance of generative artificial intelligence. Individuals who exhibit proactive personalities are more likely to take the initiative in exploring emerging technologies and evaluating their potential advantages. When interacting with generative artificial intelligence, such individuals tend to actively identify its potential benefits, including enhanced efficiency and opportunities for innovation, which in turn strengthens their perception of its usefulness. Consequently, a proactive personality exerts a positive influence on individuals’ perceived usefulness of generative artificial intelligence, thereby facilitating its acceptance. A proactive personality is not only reflected in the exploration of new technologies, but also in the rapid adaptation to and learning of emerging innovative technologies (Hsieh & Huang, 2014; Crant, 2000). For emerging technologies, individuals with proactive personalities are generally more inclined to seek help and employ other means to improve their mastery and efficiency in using things like generative artificial intelligence (Rodríguez-Ruiz et al., 2025). This positive learning attitude and the associated behavioral patterns help reduce individuals’ perception of the complexity of generative artificial intelligence, improve its perceived ease of use, and thereby enhance acceptance.

**Figure 1:**
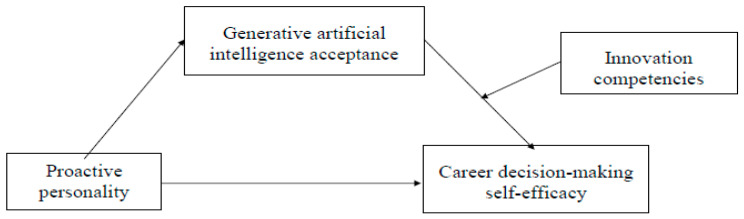
The proposed moderated mediation model.

Intrinsic motivation is a key proactive motivational state (see Parker et al., 2010; Figure 1), which has been identified as an essential driver for fostering positive attitudes toward adopting technological innovations (Hwang, 2005). This relationship is robustly anchored in the Unified Theory of Acceptance and Use of Technology (UTAUT) and its various derivatives, illustrating the interplay between personality traits and behavioral intentions concerning technology usage (Venkatesh et al., 2003, 2012). More precisely, this theory suggests that factors such as performance expectancy and effort expectancy affect technology acceptance, which represents a behavioral intention to use the technology (Venkatesh et al., 2012). In the current context, students with proactive personalities will likely be more intrinsically motivated in their assessments of generative AI, thus enhancing their performance expectancies (i.e., how they plan to use AI and what they hope to get from it) and their effort expectancies (i.e., ease of use).

Beyond these theoretical arguments, empirical research findings indicate that individuals with proactive personalities are not only more inclined toward adopting new technologies, but are also better equipped to recognize the tangible benefits associated with generative artificial intelligence (Smothers et al., 2024). These benefits include enhanced decision-making capabilities and heightened operational efficiency, both of which contribute to creating a strong rationale for technology adoption (Tariq et al., 2021). Studies conducted by Cao et al. (2021) and Kwak et al. (2022) reinforce this assertion, highlighting that proactive individuals are significantly more likely to adopt generative artificial intelligence due to their forward-thinking perspectives and an inherent understanding of potential efficiencies. In light of these findings and theoretical foundations, we propose the following hypothesis:

**Hypothesis** **2** **(H2).** *Proactive personality is positively related to generative artificial intelligence acceptance.*

### 2.3. Generative Artificial Intelligence Acceptance and Career-Related Decision-Making Self-Efficacy

As the landscape of career-related decision-making evolves in the digital age, the integration of generative artificial intelligence technologies is poised to significantly impact individuals’ self-efficacy in their career choices. The adoption of generative artificial intelligence, particularly resources like ChatGPT, is anticipated to enhance users’ self-efficacy, which refers to their belief in their capability to make effective career decisions.

When exploring generative artificial intelligence acceptance, we employ the framework of the traditional Technology Acceptance Model (TAM), which focuses on perceived usefulness and perceived ease of use as its central variables (Davis, 1989), extending its application to the domain of career-related decision-making processes. We posit that the greater an individual’s acceptance of generative artificial intelligence, the more they perceive its utility in career information searches, career planning, and other aspects, thereby enhancing their self-efficacy during the career-related decision-making process. Social cognitive theory provides support for this supposition. Within social cognitive theory, self-efficacy may be shaped through mastery experiences (Wood & Bandura, 1989), and the acceptance and use of generative artificial intelligence can serve as one means of enhancing self-perceptions of overcoming obstacles to succeed in career-related planning and preparation efforts. Indeed, research by Lent et al. (2017) and Chen et al. (2023) underscores the role of generative artificial intelligence in providing users with sophisticated capabilities for information processing and scenario analysis. These advanced capabilities are crucial in an era where career decisions are becoming increasingly complex and multifaceted.

Drawing on relevant theories related to career-related decision-making, such as the Career Decision Process Model (Gati et al., 1996), we acknowledge that the career-related decision-making process is a multifaceted information-processing task encompassing several stages, including information gathering, assessment, comparison, and selection. By utilizing generative artificial intelligence tools, individuals can enhance their decision-making processes, gaining access to a wealth of insights and analyses that were previously less accessible. The adoption of generative artificial intelligence may indirectly boost career-related decision-making self-efficacy by improving the efficiency and quality of information processing at each stage of decision-making.

Furthermore, the use of generative artificial intelligence technologies supports the exploration of diverse perspectives and comprehensive information, empowering users to critically assess their career options. This holistic approach not only refines the decision-making process, but also cultivates a stronger sense of agency and confidence in individuals as they navigate their career paths (Pordelan & Hosseinian, 2022; Akiba & Fraboni, 2023). As a result, we propose the following hypothesis:

**Hypothesis** **3** **(H3).** *Generative artificial intelligence acceptance is positively related to career-related decision-making self-efficacy.*

### 2.4. The Mediating Role of Generative Artificial Intelligence Acceptance

The notion that generative artificial intelligence acceptance serves as a mediator is consistent with the principles outlined in social cognitive theory (SCT; Bandura, 1991), which highlights the importance of self-efficacy, expected outcomes, and personal goals in career development (Lent et al., 2005). According to social cognitive theory, a college student’s personality traits influence their tendency to seek support and resources from the environment (Lent et al., 2005, 2015). Proactive personality is defined as a relatively stable tendency to effect environmental change, distinguishing individuals based on their efforts to impact their environments (Bateman & Crant, 1993; Hsieh & Huang, 2014). College students with a high degree of proactive personality do not passively accept the constraints imposed by the external environment; rather, they strive to “identify opportunities and act on them, show initiative, take action, and persevere until they bring about meaningful change” (Crant, 2000, p. 439). Proactive college students are characterized by their tendency to take initiative, anticipate future challenges, and actively seek opportunities for improvement, which makes them more likely to regard advanced technologies such as generative AI (e.g., DeepSeek or ChatGPT) as significant resources that can help them achieve their career goals.

The integration of SCT with technology acceptance models enables a deeper understanding of how proactive college students’ adoption of innovative technologies like generative artificial intelligence amplifies their personal resources, thereby strengthening their career-related decision-making self-efficacy (Lent & Brown, 2019; Lent et al., 2005, 2015; Russo, 2024). Furthermore, the Unified Theory of Acceptance and Use of Technology (UTAUT) posits that facilitating conditions, which reflect individuals’ perceptions of available resources supporting a specific behavior (Venkatesh et al., 2003), interact with behavioral intentions to use a technology, collectively determining its adoption. Within this framework, the acceptance of generative artificial intelligence (a behavioral intention) and the availability of generative AI tools such as DeepSeek (a facilitating condition) act as a joint mediating mechanism that transforms students’ proactive engagement into heightened career-related decision-making self-efficacy.

In support of these theoretical arguments, empirical evidence suggests that when proactive individuals engage with generative artificial intelligence, they utilize advanced support for information processing and scenario analysis, consequently reinforcing their confidence in making informed career decisions. This enhanced self-efficacy enables individuals to make more decisive career moves while simultaneously fostering continuous exploration and engagement with career development resources. Based on the aforementioned considerations, we propose the following hypothesis for empirical testing:

**Hypothesis** **4** **(H4).** *Generative artificial intelligence acceptance mediates the positive relationship between proactive personality and career-related decision-making self-efficacy.*

### 2.5. The Moderating Role of Innovation Competencies

Given that innovation competencies are critical in adapting to and utilizing new technologies (Kipper et al., 2021), our study investigates how these competencies potentially augment the effect of generative artificial intelligence acceptance on self-efficacy. This approach facilitates a more precise examination of how innovation competencies amplify the benefits of generative artificial intelligence, yielding valuable insights for both theoretical understanding and practical application. According to the Unified Theory of Acceptance and Use of Technology (UTAUT), “individual difference variables…are theorized to moderate various UTAUT relations” (Venkatesh et al., 2012, p. 159). We advance innovation competencies as an individual difference with a potential for moderating the relationship between generative artificial intelligence acceptance and career-related decision-making self-efficacy.

Innovation competencies encompass a blend of skills, knowledge, and capabilities that enable individuals to engage in innovative activities, effectively utilize new technologies, and generate creative solutions (Anderson et al., 2014). Innovation competencies significantly influence an individual’s confidence in managing occupational decision-making processes by providing them with the essential skills and mindset to navigate complex career environments. Empirical studies indicate that innovation competencies, particularly adaptability and problem-solving abilities, are positively associated with increased self-assurance in career-related decisions (Li et al., 2022; Stead et al., 2021; Xu et al., 2025). Higher levels of innovation competence among undergraduate students improve their employability by strengthening their confidence in their ability to perform specific tasks and achieve set objectives (Li et al., 2022). Innovation competencies encompass skills such as creative problem-solving, adaptability, and a commitment to continuous learning. These competencies are vital for effectively utilizing emerging technologies, including generative artificial intelligence (Bilgram & Laarmann, 2023). Individuals who excel in these areas are better prepared to address career-related uncertainties and make informed decisions (S. Wang et al., 2023).

The dual process theory of cognition (Evans & Stanovich, 2013; Kahneman, 2011) offers theoretical grounding for these propositions, supported by empirical evidence. As previously discussed, creative problem-solving and systems thinking are central components of innovation competencies (Keinänen et al., 2018). According to the dual process theory, individuals typically rely on either automatic, heuristic-driven thinking (Type 1) or more deliberate, analytical thinking (Type 2) when addressing complex problems. People who engage in systems thinking may more strategically leverage generative artificial intelligence technologies to enhance their decision making, while those who reply more on heuristic approaches may rely on generative artificial intelligence with little to no critical assessment. In the context of the current study, students who are high in innovative competencies are likely to engage in more deliberate and systematic thinking, which is likely to interact with their acceptance of generative artificial intelligence to enhance their career-related decision-making self-efficacy. In contrast, students with lower innovation competencies may be more likely to engage in automatic thinking, simply tending to rely on the speed and convenience of artificial intelligence, and to accept its outcomes with little critical evaluation, which could serve to undermine their career-related decision-making self-efficacy.

With the increasing adoption of generative artificial intelligence, individuals possessing robust innovation competencies are better positioned to leverage these technologies for enhanced performance and decision-making. Individuals exhibiting high innovation competencies are more likely to recognize the potential of generative artificial intelligence technologies and integrate them effectively, subsequently enhancing their self-efficacy. Conversely, individuals with less developed innovation competencies may encounter difficulties in recognizing the value of generative artificial intelligence technologies, potentially leading to diminished self-efficacy and suboptimal career decision outcomes. Notwithstanding the significance of this relationship, the extant literature has not comprehensively examined how innovation competencies modulate the impact of a proactive personality on self-efficacy in career-related decision-making via generative artificial intelligence acceptance. Consequently, based on these theoretical and logical arguments, we propose the following hypotheses:

**Hypothesis** **5** **(H5).** *Innovation competencies moderate the positive relationship between generative artificial intelligence acceptance and career-related decision-making self-efficacy, such that the relationship is stronger for college students with higher innovation competencies compared to those with low competencies.*

**Hypothesis** **6** **(H6).** *Innovation competencies moderate the mediating effect of proactive personality on career-related decision-making self-efficacy via generative artificial intelligence acceptance, such that the indirect effect is stronger when students’ innovation competencies are higher compared to when they are low.*

To summarize, our moderated mediation model is shown in Figure 1.

## 3. Research Method

### 3.1. Participants and Procedures

This study focused on college students as the target population. Convenience sampling was utilized to conduct a questionnaire-based survey among students enrolled at universities in Guangdong Province from November to December 2024. The surveys were completed using an electronic mobile survey platform that allowed participants to submit their responses at varied times and places. To reduce common method bias, we collected the data at four times, separated by one week each (Podsakoff et al., 2012). At time 1, the participants completed measures of proactive personality and demographic variables. At time 2, one week later, the participants were assessed for generative artificial intelligence acceptance. At time 3, which occurred one week after time 2, the participants were assessed for their level of innovation competencies. At time 4, which occurred one week after time 3, the participants completed measures of career-related decision-making self-efficacy. A total of 578 questionnaires were distributed, with 526 received and 501 deemed valid, yielding an effective response rate of 86.67%.

The survey process adhered to rigorous confidentiality protocols, safeguarding participants’ privacy throughout the study’s duration. Informed consent was obtained from all participants prior to their involvement in the study. The research design and implementation fully complied with the ethical standards delineated in the Helsinki Declaration. Participants were provided with explicit instructions concerning the study’s research purposes, item completion protocols, the voluntary nature of their participation, and assurances of anonymity.

### 3.2. Measures

#### 3.2.1. Proactive Personality

This study employed the Chinese adaptation of the Proactive Personality Scale (Shang & Gan, 2009), originally developed by Bateman and Crant (1993), to assess proactive personality. The scale consists of 11 items measured on a 7-point Likert-type scale, ranging from 1 (“strongly disagree”) to 7 (“strongly agree”). Higher scores indicate a higher level of proactive personality. Considering the specific context of the participants and the study’s requirements, 6 items from the original scale were excluded after careful deliberation, leaving 5 items for the final measure. A sample item from this scale is: “When faced with a problem, I confront it directly”. The revised scale showed excellent internal consistency Cronbach’s α = 0.91. Confirmatory factor analysis revealed standardized factor loadings ranging from 0.57 to 0.77, all exceeding the 0.5 threshold, indicating the robust reliability and validity of the measurement. The model fit indices (*χ*^2^*/df* = 2.50, RMSEA = 0.06, SRMR = 0.03, CFI = 0.96, and TLI = 0.95) indicate a good fit to the data (Schumacker & Lomax, 2004; Bollen, 1989).

#### 3.2.2. Generative Artificial Intelligence Acceptance

Generative artificial intelligence acceptance was measured using the Generative Artificial Intelligence Acceptance Scale (Yilmaz et al., 2024). The scale consists of four dimensions: performance expectation, effort expectation, facilitating conditions, and social influence. It originally included 20 items measured on a 5-point Likert-type scale, ranging from 1 (“strongly disagree”) to 5 (“strongly agree”). Higher scores indicate a higher level of acceptance of generative artificial intelligence. Considering the specific context of the participants and the study’s objectives, 5 items from the social influence dimension were excluded, resulting in a 15-item scale comprising the remaining three dimensions. A sample item from the scale is: “I find generative artificial intelligence applications useful in my daily life”. The Cronbach’s alpha coefficient for the adapted scale in this study was 0.93. The standardized factor loadings, revealed through confirmatory factor analysis, ranged from 0.70 to 0.80, all exceeding the 0.5 threshold, thus demonstrating the strong reliability and validity of the measurement. The model fit indices (*χ*^2^*/df* = 2.28, RMSEA = 0.05, SRMR = 0.03, CFI = 0.98, and TLI = 0.98) indicate a good fit between the data and the model (Schumacker & Lomax, 2004; Bollen, 1989).

#### 3.2.3. Innovation Competencies

Innovation Competencies were measured using the 12-item scale developed by Keinänen et al. (2018). The adapted scale focuses on two key dimensions: creative problem-solving and systems thinking. Each item was assessed using a Likert-type response scale ranging from 1 (“none”) to 5 (“excellent”). A sample item is that “I suggest new practical solutions to reach a goal”. The Cronbach’s alpha coefficient for the adapted scale in this study was 0.93. The standardized factor loadings, revealed through confirmatory factor analysis, ranged from 0.70 to 0.80, all exceeding the 0.5 threshold, thus demonstrating the strong reliability and validity of the measurement. The model fit indices (*χ*^2^/*df* = 2.28, RMSEA = 0.05, SRMR = 0.03, CFI = 0.98, and TLI = 0.98) indicate a good fit between the data and the model (Schumacker & Lomax, 2004; Bollen, 1989).

#### 3.2.4. Career Decision-Making Self-Efficacy

The Career Decision-Making Self-Efficacy Scale, originally developed by Betz et al. (1996), was adapted into Chinese through successive revisions by Peng and Long (2001). For this study, three dimensions—information gathering, goal setting, and problem-solving—were retained from the original scale to better align with the characteristics of the participants and the study’s objectives. The adapted scale comprises 9 items, each assessed on a 5-point Likert scale, ranging from 1 (“very unconfident”) to 5 (“very confident”). Higher scores denote a greater perceived self-efficacy in career-related decision-making. A sample item from this scale is, “How confident are you in identifying the profession or job you are most capable of performing?” The Cronbach’s alpha coefficient for the adapted scale in this study was 0.87. The standardized factor loadings, as revealed by confirmatory factor analysis, ranged from 0.59 to 0.82, all exceeding the 0.5 threshold, thus demonstrating the strong reliability and validity of the measurement. The model fit indices (*χ*^2^/*df* = 2.43, RMSEA = 0.05, SRMR = 0.03, CFI = 0.98, and TLI = 0.97) indicate an excellent fit between the data and the model (Schumacker & Lomax, 2004; Bollen, 1989).

## 4. Results

### 4.1. Common Method Bias Testing

A confirmatory factor analysis (CFA) was conducted to assess the discriminant validity of the four latent variables under investigation. As presented in Table 1, the results indicate that the four-factor model showed the best fit to the data, with key fit indices supporting its robustness. Specifically, the Root Mean Square Error of Approximation (RMSEA) was 0.05, and the Standardized Root Mean Square Residual (SRMR) was 0.04, both falling within the acceptable ranges indicative of a well-fitting model. Furthermore, the Comparative Fit Index (CFI) and Tucker–Lewis Index (TLI) were both 0.92. Based on model fit evaluation recommendations (Kline, 2011), the hypothesized four-factor model fit the data better than the alternative models.

### 4.2. Descriptive Statistics and Correlation Analyses

The sample consisted of 240 males (47.9%) and 261 females (52.1%), with participants distributed across academic levels: 132 freshmen (26.3%), 194 sophomores (38.7%), 100 juniors (20.0%), and 75 seniors (15.0%). N = 501. Descriptive statistics, including means (M), standard deviations (SD), and Pearson correlation coefficients, were computed for the main research variables. As shown in Table 2, significant positive correlations were observed between proactive personality (PP) and generative AI acceptance (GAIA) (*r* = 0.58, *p* < 0.01), innovation competencies (IC) (*r* = 0.62, *p* < 0.01), and career decision-making self-efficacy (CDMSE) (*r* = 0.59, *p* < 0.01). Similarly, GAIA was positively correlated with IC (*r* = 0.66, *p* < 0.01) and CDMSE (*r* = 0.64, *p* < 0.01). Additionally, IC showed a strong positive correlation with CDMSE (*r* = 0.69, *p* < 0.01). These results provide robust empirical support for the hypothesized relationships. Gender and academic level were controlled for in subsequent analyses.

### 4.3. Direct Effect Testing

We tested the direct effects between variables using linear regression in SPSS 26.0. As shown in Table 3, proactive personality was significantly associated with generative artificial intelligence acceptance (*β* = 0.574, *t* = 15.789, *p* < 0.001). Additionally, proactive personality positively predicted career-related decision-making self-efficacy (*β* = 0.575, *t* = 16.244, *p* < 0.001). In Table 4, generative artificial intelligence acceptance positively influenced career-related decision-making self-efficacy (*β* = 0.623, *t* = 18.341, *p* < 0.001). Therefore, these findings support Hypotheses 1–3.

### 4.4. Mediation Effect Testing

To examine the mediation effect, we employed Model 4 from the PROCESS macro (Hayes, 2017) in SPSS. A mediation analysis was conducted with proactive personality as the independent variable, career-related decision-making self-efficacy as the dependent variable, and generative artificial intelligence acceptance as the mediator. As shown in Table 5, proactive personality positively predicted generative artificial intelligence acceptance (*β* = 0.574, *t* = 15.789, *p* < 0.001), which supports Hypothesis 1. Additionally, both proactive personality and generative artificial intelligence acceptance had significant effects on career-related decision-making self-efficacy (*β* = 0.325, t = 8.379, *p* < 0.001; *β* = 0.434, *t* = 11.146, *p* < 0.001). This provides preliminary evidence that proactive personality influences career-related decision-making self-efficacy through the mediation of generative artificial intelligence acceptance. However, further verification using the Bootstrap method is required to confirm the mediating effect. Bootstrapping with 5000 resamples was used to estimate the 95% confidence intervals (CIs) for the direct and indirect effects. The CIs for both effects exclude zero. These results suggest that generative artificial intelligence acceptance partially mediates the relationship between proactive personality and career-related decision-making self-efficacy. Specifically, the direct effect (0.325) accounted for 56.522% of the total effect, while the indirect effect (0.250) through the mediator contributed 43.478% (see Table 6). Therefore, these findings support Hypothesis 4.

### 4.5. Moderated Mediation Effect Testing

To test Hypotheses 5 and 6, we examined the second-stage moderation in the mediation model by incorporating innovation competency as a moderating variable. We used Model 14 from the PROCESS macro (Hayes, 2017) in SPSS to assess the moderated mediation model.

As shown in Table 7, the initial regression model predicting generative artificial intelligence acceptance from proactive personality showed a strong and significant effect, with proactive personality explaining 33.3% of the variance in generative artificial intelligence acceptance (*R*^2^ = 0.333, *F* = 249.282, *p* < 0.001). The standardized regression coefficient (*β*) for proactive personality was 0.574, indicating a substantial direct effect on generative artificial intelligence acceptance (*β* = 0.574, *t* = 15.789, *p* < 0.001). Hypothesis 1 is thus supported again.

In the second regression model predicting career-related decision-making self-efficacy from proactive personality, generative artificial intelligence acceptance, innovation competency, and the interaction term between generative artificial intelligence acceptance and innovation competency, the overall model was highly significant, accounting for 58.5% of the variance in career-related decision-making self-efficacy (*R*^2^ = 0.585, *F* = 174.985, *p* < 0.001). The direct effect of proactive personality on career-related decision-making self-efficacy was significant but relatively small (*β* = 0.195, *t* = 5.160, *p* < 0.001). Generative artificial intelligence acceptance had a substantial direct effect on career-related decision-making self-efficacy (*β* = 0.288, *t* = 7.287, *p* < 0.001), and innovation competency also had a significant direct effect on career-related decision-making self-efficacy (*β* = 0.320, *t* = 7.355, *p* < 0.001). The interaction term between generative artificial intelligence acceptance and innovation competency was significant (*β* = 0.189, *t* = 6.247, *p* < 0.001), indicating that the effect of generative artificial intelligence acceptance on career-related decision-making self-efficacy was moderated by innovation competency.

**Figure 2:**
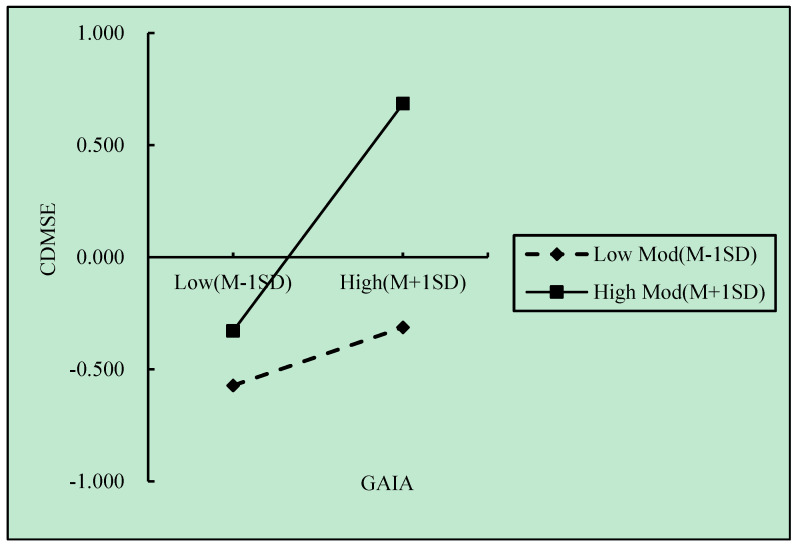
Test of the moderation effect, revealing how IC influences the association connecting GAIA and CDMSE.

The interaction effect between generative artificial intelligence acceptance and innovation competency was further explored to understand the conditional effects of generative artificial intelligence acceptance on career-related decision-making self-efficacy at different levels of innovation competency. Table 8 shows that the effect of generative artificial intelligence acceptance on career-related decision-making self-efficacy varied significantly depending on the level of innovation competency. Specifically, when innovation competency was one standard deviation below the mean, the effect of generative artificial intelligence acceptance on career-related decision-making self-efficacy was smaller (*β* = 0.108, *p* < 0.05). At the mean level of innovation competency, the effect of generative artificial intelligence acceptance on career-related decision-making self-efficacy was moderate (*β* = 0.288, *p* < 0.001). When innovation competency was one standard deviation above the mean, the effect of generative artificial intelligence acceptance on career-related decision-making self-efficacy was stronger (*β* = 0.468, *p* < 0.001). This pattern suggests that innovation competency significantly moderated the relationship between generative artificial intelligence acceptance and career-related decision-making self-efficacy. The positive effect of generative artificial intelligence acceptance on career-related decision-making self-efficacy was significant across all levels of innovation competencies, with the effect strengthening as innovation competency increased (visualized in Figure 2). Hypothesis 5 is thus supported.

To further examine the moderated mediation effect, we conducted a simple slopes analysis, with innovation competencies categorized into low (M − 1 SD) and high (M + 1 SD) levels. The results indicate that the indirect effect of proactive personality on career-related decision-making self-efficacy through generative artificial intelligence acceptance was moderated by innovation competencies. The conditional indirect effect was significant at all levels of innovation competencies (low IC—effect = 0.062, 95%CI [0.009, 0.117]; medium IC—Effect = 0.165, 95%CI [0.113, 0.222]; high IC—effect = 0.269, 95%CI [0.202, 0.341]).

The difference in the indirect effect between high and low levels of innovation competencies was 0.207 (95%CI [0.147, 0.269]). The index of moderated mediation (Hayes, 2017) was 0.109 (95%CI [0.077, 0.141]), confirming the presence of a moderated mediation effect (see Table 9). These findings provide support for Hypothesis 6.

## 5. Discussion

### 5.1. Summary of Findings

This research has investigated how proactive personality is associated with career-related decision-making self-efficacy, focusing specifically on the mediating role of generative artificial intelligence acceptance and the moderating influence of innovation competencies. The findings suggest that proactive personality is positively related to career-related decision-making self-efficacy, and that this relationship is transmitted indirectly via the acceptance of generative artificial intelligence. This suggests that individuals exhibiting proactive qualities are more likely to adopt new technologies, which thereby enhances their confidence in making vocational choices.

The study’s findings show that there is an important role that innovation competencies play, enhancing this mediation effect in a significant manner. This means that when people work on their innovation ability, the relation between generative artificial intelligence acceptance and occupational decision-making confidence will become stronger. These situations point out that a proactive personality and also technological acceptance play roles that are not small when it comes to how career choices are effectively made, especially because of how fast the job market is now changing and developing in different ways.

These findings are in agreement with previous research that had already pointed out how having a proactive personality is important and connected to having better career growth (Kim & Park, 2017; Lent et al., 2017). But this research at present takes one step further by putting more focus on how the acceptance of generative artificial intelligence and competencies of innovation play important roles in such a situation. Bringing these factors together not only makes it more clear to see how a proactive personality can bring different results, but it also makes it more understandable what specific key aspects are more relevant, especially when technology is advancing more and more. Such an approach makes people see other possible future research ideas, and also ways in which this could be useful for practice in areas related to developing careers.

### 5.2. Interpretation of Results

The results of this study highlight the crucial importance of a proactive personality in the context of vocational decision-making. Individuals with a proactive disposition are more likely to actively engage with and adopt innovative technological advancements, such as generative artificial intelligence. This tendency is particularly noteworthy because it enhances their confidence in making well-informed career decisions. The significant positive correlation observed between the acceptance of generative artificial intelligence and occupational decision-making confidence suggests that artificial intelligence tools can be highly valuable resources for students. These tools provide access to extensive information and offer decision-making support, thereby enabling users to more effectively navigate their career paths.

The findings are consistent with the general principle that proactive individuals tend to be more open to new experiences and technologies. This openness enables them to incorporate innovative resources into their decision-making processes, and enhances their self-efficacy when facing complex tasks, such as career planning (Zhou et al., 2018; Pordelan & Hosseinian, 2022).

The impact of innovation competencies being moderate reveals more layers of findings. People with a higher level of innovation competency find themselves in a much better place to use the advantages that generative artificial intelligence brings. This enhances self-efficacy in career-related decision-making overall. The research results suggest that innovation competencies in college students, such as the ability to solve problems creatively and adapt to dynamic situations, are very critical for navigating modern careers (Keinänen et al., 2018; Bilgram & Laarmann, 2023). Innovation competencies not only help college students improve their skills in using new technologies, but also enable them to deal with difficulties and challenges while maximizing opportunities for career development.

In conclusion, the insights derived from this study highlight the necessity of fostering both proactive mindsets and innovation competencies among students. This effort will not only provide them with the necessary tools for effective career-related decision-making, but also prepare them for the rapidly changing technological environment that defines contemporary workplaces.

### 5.3. Theoretical Contributions

This study makes significant contributions to the existing literature in several key areas. First, it comprehensively integrates active personality theory, technology acceptance models, and social cognitive career theory. This synthesis enhances our understanding of the complex interplay between personality traits and technology acceptance, especially in the context of emerging AI technologies. By placing these concepts within a unified framework, this study illuminates how a proactive personality enhances technology acceptance, which in turn influences career-related outcomes (Bandura, 1977; Bateman & Crant, 1993; Venkatesh et al., 2012).

Second, the study offers evidence that is empirical in nature, which supports the mixing of concepts from social cognitive career theory with models for accepting technology. This meeting point makes clear the important role that is played by technologies designed to be supportive, for example, AI that generates things. Such technology helps significantly with boosting one’s belief in one’s own career choices, which then helps shape where one’s career ends up going (according to Cao et al., 2021).

Third, innovation competencies, when added as a moderating factor, make it possible to see more deeply into the way career-related decision-making self-efficacy is affected, which is important in today’s digital times where technology is moving fast. The development of innovation competencies is something that must be sought, as technology keeps advancing rapidly. In earlier models, it was mostly either personality traits or technology acceptance that were considered, but what this study does is combine these and show that innovation competencies can make proactive tendencies even more beneficial. This brings about a different way of looking at things, whereby having a proactive personality by itself is not enough at all. If innovation competencies are also there, then the full potential of new technological changes, like AI systems that generate content, can be used properly in making job-related choices.

Last but not least, the social cognitive career theory has always said that when people make career decisions, things like self-efficacy, expected results, and personal goals matter a lot. However, when innovation skills are also considered, the study talks about how they do not just help people with technology, but also change how confident they feel about their career abilities. This idea means it is possible to develop a more detailed way to look at how people may be given power by getting better at skills, especially when technology is changing as fast as it is today.

### 5.4. Practical Implications

Our findings have many practical implications that can be applied in different ways. First, educational institutions need to pay attention to developing behaviors that are more proactive and innovation-related competencies that are important inside their programs. By undertaking programs that are designed for growing these skills, students can develop more ways to connect better with new digital innovations, for example, generative artificial intelligence systems. Workshops and seminars can be implemented to also bring about experiential learning opportunities, which is also helpful for developing an innovative way of thinking and for improving proactive methods to plan careers.

Second, it is important that career counseling services, in some way, incorporate training that could emphasize the use of generative artificial intelligence as an important tool when it comes to making decisions related to careers. It should be ensured that students are guided on how they might be able to use AI tools in a way that is effective, and counselors would be helping students understand how these tools can be useful, which could lead to them making better decisions about their careers. The training itself could involve having some sort of practical, hands-on experience with AI platforms that are available, platforms which may give some sort of insight or advice about careers, so that students can become more confident, and perhaps also more capable, when using technology to navigate their professional paths. The confidence, when built through experience, would hopefully help them in using these resources to make career-related decisions in a way that makes sense for their futures.

Third, peer mentorship programs need to be brought in by institutions, where students could be linked with alumni or upperclassmen who, at some time in the past, went through the same career choices. The value of these connections is in giving helpful insights about how generative artificial intelligence tools are used in a way that helps students learn skills and also feel more confident when making career choices.

Fourth, when career development courses are made to incorporate technological literacy and innovation abilities, there are positive educational effects that can happen. If such results of education are aligned with how the job market keeps changing over time, then institutions can prepare students in a way that is more suited for whatever career difficulties might arise later.

### 5.5. Limitations and Future Research Directions

It must be noted that this research does add to the understanding of how the adoption of generative artificial intelligence affects career-related decision-making self-efficacy. However, several limitations should be mentioned, which have been found to affect such studies in general.

First, the reliance on self-reported data could lead to social desirability bias. This means that it is possible that respondents, instead of providing truthful and precise responses, may have chosen to give answers that are more socially accepted or viewed positively by others. The concern about this bias has already been highlighted by Podsakoff et al. (2003). This kind of issue, if present, could possibly distort the results, and it might make it hard to accurately interpret the relationships that were examined in the study, particularly in relation to the variables involved.

Second, the cross-sectional nature of this study limits the ability to establish causal relationships. While correlations can be identified, inferring directionality or causality remains challenging due to the static nature of the data collection. Although we have presented causal arguments based on theoretical and empirical foundations, future research would benefit from longitudinal studies to help establish the causal pathways through which proactive personality traits influence the adoption of generative artificial intelligence and career-related decision-making self-efficacy. By collecting data over extended time periods, researchers can elucidate the dynamics and temporal sequences of these variables, thereby providing more robust conclusions regarding their interrelationships (Lent et al., 2017).

Third, the sample of the study was recruited from a university in Guangdong Province, China, which could bring certain biases into the findings. This may be seen as a limitation. It is known that cultural and economic situations, as well as educational environments, can differ quite a lot across regions, or even among different groups of people. Therefore, it is not completely clear how well the findings might apply beyond this particular sample. Consequently, the scope of the results might be limited by this regional focus, because the context of Guangdong might not represent what happens in other places. In future research, it could be beneficial to select a more varied and broader sample, which would allow for more general findings, and make it possible to confirm if these relationships hold true in different settings, with people from various backgrounds, or cultural situations.

Fourth, much like other research studies exploring hypothesized models, our findings and interpretations are susceptible to concerns relating unmeasured variables. Unmeasured variable problems stem from the possibility that one or more possible causal mechanism is excluded from a conceptual model, allowing for alternative explanations of the findings derived from model testing (James, 1991). While we focus on proactive personality as the key antecedent, our model does not directly measure the possible effects of fundamental dispositional factors such as general intelligence or prior academic achievement records on AI acceptance. Although meta-analytic evidence suggests that personality traits (e.g., proactivity) and cognitive ability are only weakly correlated (Judge et al., 2002), their potential confounding effects cannot be entirely ruled out. Future research could incorporate standardized cognitive tests (e.g., Raven’s Progressive Matrices) or historical performance data (e.g., GPA) to disentangle the unique effects of personality versus intelligence on AI acceptance. In addition, our model does not evaluate the effects of the heterogeneity in trust in AI. Evidence shows that individuals’ trust levels significantly shape their risk perceptions of AI adoption and long-term usage intentions (Glikson & Woolley, 2020). For example, high-trust individuals may underestimate ethical risks, thereby aggressively integrating AI into career plans, whereas low-trust counterparts might restrain career exploration due to safety concerns, even if they possess strong AI skills (Zhu et al., 2025). This trust-driven heterogeneity, unmeasured in our model, could bias path coefficient estimates. We recommend that future studies adopt multidimensional trust scales to compare mechanisms across trust levels (Gulati et al., 2019).

Fifth, the exclusive reliance on self-reported data, though methodologically justified for assessing subjective constructs like AI acceptance and career decision self-efficacy, may introduce illusory belief biases. Specifically, students’ self-claimed generative AI skills might overestimate their actual technical competence. This limitation, however, is mitigated by two considerations: (a) the UTAUT framework prioritizes perceived ease of use over objective usability in predicting behavioral intentions (Venkatesh et al., 2003), aligning with our focus on psychological adoption readiness; (b) prior meta-analyses confirm that self-efficacy scales exhibit moderate-to-strong predictive validity for real-world career behaviors (Stajkovic & Luthans, 1998). To advance causal inference, future studies should adopt multi-method evaluation, for example, combining AI task-based assessments (e.g., coding challenges with ChatGPT or DeepSeek), peer evaluations of career adaptability, and longitudinal tracking of employment outcomes.

Finally, our study does not systematically examine the differences in AI proficiency among college students and their potential moderating effects. For instance, STEM students may experience earlier exposure to generative AI tools through coding training, resulting in significant skill disparities compared to humanities majors (Nguyen et al., 2021; Parviz, 2024). These prior experience gaps could bias the assessment of career efficacy. Although we statistically controlled for the disciplinary categories (STEM vs. non-STEM) of the college students, the lack of direct measurement of actual AI competencies (e.g., through skill tests or task-based experiments) limits the generalizability of our findings. Future research should integrate objective proficiency metrics (e.g., AI project completion rates) related to college students to enhance this study.

## 6. Conclusions

Proactive personality was found to be indirectly related to career-related decision-making self-efficacy through the acceptance of generative artificial intelligence. Notably, innovation competencies moderated this mediation effect, wherein the relationship between proactive personality and career-related decision-making self-efficacy is amplified at higher levels of innovation competencies. These findings contribute to a more comprehensive and nuanced understanding of the positive relationship between proactive personality and career-related decision-making self-efficacy.

The integration of proactive personality traits and innovation competencies, and the acceptance of generative artificial intelligence, presents a promising approach to enhancing students’ career-related decision-making self-efficacy. Educational programs that cultivate these traits and skills, while concurrently promoting the effective utilization of generative artificial intelligence tools, have the potential to better equip students for success in an increasingly complex and technologically driven labor market.

In conclusion, this study emphasizes the significance of fostering a proactive disposition and innovation competencies in students, while simultaneously promoting the integration and utilization of generative artificial intelligence as a resource for career development. These findings contribute to the body of knowledge regarding how students can be effectively supported in their career-related decision-making processes, ultimately facilitating their navigation of employment challenges with enhanced self-efficacy and proficiency.

## Figures and Tables

**Table 1:** The result of confirmatory factor analyses.

Model	*χ* ^2^	*df*	*χ*^2^/*df*	RMSEA	SRMR	CFI	TLI
Four-factor model ^a^	1624.65	773	2.10	0.05	0.04	0.92	0.92
Three-factor model ^b^	2167.46	776	2.79	0.06	0.06	0.87	0.86
Three-factor model ^c^	2236.07	776	2.88	0.06	0.05	0.86	0.86
Three-factor model ^d^	2514.31	776	3.24	0.07	0.06	0.84	0.83
Two-factor model ^e^	2778.70	778	3.57	0.07	0.06	0.81	0.80
One-factor model ^f^	3421.93	779	4.39	0.08	0.07	0.75	0.74

N = 501. PP: proactive personality. GAIA: generative artificial intelligence acceptance. IC: innovation competencies. CDMSE: career-related decision-making self-efficacy. ^a^ PP, GAIA, IC, CDMSE; ^b^ PP, IC, GAIA + CDMSE; ^c^ PP + IC, GAIA, CDMSE; ^d^ PP, IC + GAIA, CDMSE; ^e^ PP + IC, GAIA + CDMSE; ^f^ PP + IC + GAIA + CDMSE. The symbol “+” denotes the combination of factors.

**Table 2:** Descriptive statistics and correlations among all variables.

Variable	M	SD	1	2	3	4	5	6	7
1. Gender ^a^	0.52	0.50	—						
2. Grades	2.24	1.00	−0.06	—					
3. Major ^b^	1.49	0.50	0.08	−0.18 **	—				
4. PP	5.16	1.01	−0.14 **	0.03	0.04	—			
5. GAIA	3.92	0.49	−0.15 **	−0.00	0.07	0.58 **	—		
6. IC	3.7	0.59	−0.16 **	−0.01	0.10 *	0.62 **	0.66 **	—	
7. CDMSE	3.69	0.44	−0.21 **	−0.05	0.07	0.59 **	0.64 **	0.69 **	—

N = 501; * *p* < 0.05, ** *p* < 0.01. PP: proactive personality. GAIA: generative artificial intelligence acceptance. IC: innovation competencies. CDMSE: career decision-making self-efficacy. ^a^ 0 = male, 1 = female; ^b^ 1 = STEM, 2 = non-STEM.

**Table 3:** Testing the direct effects of PP on GAIA and PP on CDMSE.

Predictors			On GAIA				On CDMSE	
	*β*	*SE*	*t*	95%CI	*β*	*SE*	*t*	95%CI
Constant	0.007	0.036	0.191	[−0.063, 0.077]	−0.064	0.035	−1.854	[−0.132, 0.004]
PP	0.574	0.036	15.789 ***	[0.503, 0.646]	0.575	0.035	16.244 ***	[0.505, 0.644]
*R* ^2^	0.333				0.346			
*F*	249.282 ***				263.851 ***			

N = 501; *** *p* < 0.001. CI: confidence interval. PP: proactive personality. GAIA: generative artificial intelligence acceptance. CDMSE: career decision-making self-efficacy.

**Table 4:** Testing the direct effect of GAIA on CDMSE.

Predictors			On CDMSE	
	*β*	*SE*	*t*	95%CI
Constant	−0.060	0.033	−1.825	[−0.125, 0.005]
GAIA	0.623	0.034	18.341 ***	[0.556, 0.690]
*R* ^2^	0.403			
*F*	336.375 ***			

N = 501; *** *p* < 0.001. CI: confidence interval. GAIA: generative artificial intelligence acceptance. CDMSE: career decision-making self-efficacy.

**Table 5:** Testing the mediating effect of PP on CDMSE.

Predictors			On GAIA				On CDMSE	
	*β*	*SE*	*t*	95%CI	*β*	*SE*	*t*	95%CI
Constant	0.007	0.036	0.191	[−0.063, 0.077]	−0.067	0.031	−2.165 *	[−0.128, −0.006]
PP	0.574	0.036	15.789 ***	[0.503, 0.646]	0.325	0.039	8.379 ***	[0.249, 0.401]
GAIA					0.434	0.039	11.146 ***	[0.358, 0.511]
*R* ^2^	0.333				0.476			
*F*	249.282 ***				226.620 ***			

N = 501; * *p* < 0.05, *** *p* < 0.001. CI: confidence interval. PP: proactive personality. GAIA: generative artificial intelligence acceptance. CDMSE: career decision-making self-efficacy.

**Table 6:** Total effect, direct effect, and indirect effect among the variables.

	Effect	Boot SE	Boot CI	Boot CI	Relative
	Size		Lower	Upper	Effect Size
			Limit	Limit	
Total effect	0.575	0.035	0.505	0.644	
Direct effect	0.325	0.039	0.249	0.401	56.522%
Indirect effect	0.250	0.030	0.195	0.311	43.478%

N = 501; CI: confidence interval.

**Table 7:** Testing the moderated mediating effect of PP on CDMSE.

Predictors			On GAIA				On CDMSE	
	*β*	*SE*	*t*	95%CI	*β*	*SE*	*t*	95%CI
Constant	−0.021	0.036	−0.599	[−0.091, 0.049]	−0.165	0.033	−5.011 ***	[−0.230, −0.100]
PP	0.574	0.036	15.789 ***	[0.503, 0.646]	0.195	0.038	5.160 ***	[0.121, 0.269]
GAIA					0.288	0.040	7.287 ***	[0.210, 0.366]
IC					0.320	0.043	7.355 ***	[0.234, 0.405]
GAIA × IC					0.189	0.029	6.427 ***	[0.131, 0.247]
*R* ^2^	0.333				0.585			
*F*	249.282 ***				174.985 ***			

N = 501; *** *p* < 0.001. CI: confidence interval. PP: proactive personality. GAIA: generative artificial intelligence acceptance. IC: innovation competencies. CDMSE: career decision-making self-efficacy.

**Table 8:** Conditional effects of GAIA at values of IC.

IC	Effect	*SE*	*t*	95%CI
Low (M − 1 *SD*)	0.108	0.046	2.336 *	[0.017, 0.198]
Medium (*M*)	0.288	0.040	7.287 ***	[0.210, 0.366]
High (M + 1 *SD*)	0.468	0.051	9.224 ***	[0.369, 0.568]

N = 501; * *p* < 0.05, *** *p* < 0.001. CI: confidence interval. GAIA: generative artificial intelligence acceptance. IC: innovation competencies.

**Table 9:** Conditional indirect effect at specific levels of IC when mediated by GAIA.

Conditional Indirect Effect of IC	Effect	Boot *SE*	95%CI
Low (M − 1 *SD*)	0.062	0.027	[0.009, 0.117]
Medium (*M*)	0.165	0.027	[0.113, 0.222]
High (M + 1 *SD*)	0.269	0.035	[0.202, 0.341]
Difference between indirect effect	0.207	0.031	[0.147, 0.269]
Index of moderated mediation	0.109	0.016	[0.077, 0.141]

N = 501; CI: confidence interval. GAIA: generative artificial intelligence acceptance. IC: innovation competencies.

## Data Availability

The manuscript encompasses all relevant data that support the findings of this study. For further information or queries, interested parties may contact the corresponding author.
